# Case Report: Characterizing the Role of the STXBP2-R190C Monoallelic Mutation Found in a Patient With Hemophagocytic Syndrome and Langerhans Cell Histiocytosis

**DOI:** 10.3389/fimmu.2021.723836

**Published:** 2021-09-23

**Authors:** Laura Viñas-Giménez, Rafael Rincón, Roger Colobran, Xavier de la Cruz, Verónica Paola Celis, José Luis Dapena, Laia Alsina, Joan Sayós, Mónica Martínez-Gallo

**Affiliations:** ^1^ Immunology Division, Hospital Universitari Vall d’Hebron (HUVH), Jeffrey Model Foundation Excellence Center, Barcelona, Spain; ^2^ Diagnostic Immunology Research Group Vall d’Hebron Research Institute (VHIR), Barcelona, Spain; ^3^ Department of Cell Biology, Physiology and Immunology, Autonomous University of Barcelona (UAB), Barcelona, Spain; ^4^ Genetics Department, Hospital UniversitariVall d’Hebron (HUVH), Barcelona, Spain; ^5^ Research Unit in Translational Bioinformatics in Neurosciences, Universitat Autònoma de Barcelona, Barcelona, Spain; ^6^ Institut Catala per la Recerca i Estudis Avançats (ICREA), Barcelona, Spain; ^7^ Hematology Department, Pediatric Cancer Center, Hospital Sant Joan de Déu, Esplugues de Llobregat, Spain; ^8^ Clinical Immunology and Primary Immunodeficiencies Unit, Allergy and Clinical Immunology Department, Hospital Sant Joan de Déu, University of Barcelona, Pediatric Research Institute Sant Joan de Déu, Barcelona, Spain; ^9^ Immune Regulation and Immunotherapy Group, CIBBIM-Nano medicine, Vall d’Hebron Institut de Recerca, Universitat Autonoma de Barcelona, Barcelona, Spain

**Keywords:** HLH, STXBP2, Langerhans-cell-histiocytosis, degranulation, NK, familial hemophagocytic lymphohistiocytosis−5 (f-HLH), lytic granule exocytosis, STX11

## Abstract

Hemophagocytic lymphohistiocytosis (HLH) is a life-threatening hyperinflammatory disorder. HLH can be considered as a threshold disease depending on the trigger and the residual NK-cell cytotoxicity. In this study, we analyzed the molecular and functional impact of a novel monoallelic mutation found in a patient with two episodes of HLH. A 9-month-old child was diagnosed at 2 months of age with cutaneous Langerhans cell histiocytosis (LCH). After successful treatment, the patient developed an HLH episode. At 16 month of age, the patient went through an HSCT losing the engraftment 5 months later concomitant with an HLH relapse. The genetic study revealed a monoallelic mutation in the STXBP2 gene (.pArg190Cys). We transfected COS7 cells to analyze the STXBP2-R190C expression and to test the interaction with STX11. We used the RBL-2H3 cell line expressing STXBP2-WT-EGFP or R190C-EGFP for degranulation assays. Mutation STXBP2-R190C did not affect protein expression or interaction with syntaxin-11. However, we have demonstrated that STXBP2-R190C mutation diminishes degranulation in the RBL-2H3 cell line compared with the RBL-2H3 cell line transfected with STXBP2-WT or nontransfected. These results suggest that STXBP2-R190C mutation acts as a modifier of the degranulation process producing a decrease in degranulation. Therefore, under homeostatic conditions, the presence of one copy of STXBP2-R190 could generate sufficient degranulation capacity. However, it is likely that early in life when adaptive immune system functions are not sufficiently developed, an infection may not be resolved with this genetic background, leading to a hyperinflammation syndrome and eventually develop HLH. This analysis highlights the need for functional testing of new mutations to validate their role in genetic susceptibility and to establish the best possible treatment for these patients.

## Introduction

Hemophagocytic lymphohistiocytosis (HLH) is a multisystem hyperinflammatory syndrome characterized by persistent macrophages/lymphocytes activation. Clinical diagnosis of HLH is challenging and requires compliance with 5/8 clinical and laboratory criteria or a defined molecular diagnosis.

HLH has been classified classically into familial/primary (FHL) and acquired/secondary. FHL is caused by mutations in several genes encoding proteins of the cytotoxic pathway. Secondary HLH is considered in those patients without genetic diagnosis or family history and typically appear in the context of infection or malignancy or in a variety of inflammatory and autoimmune conditions with immune activation. In the traditional classification, only individuals harboring biallelic germline mutations are formerly typified as FHL. Recent reports show patients developing a clinical picture of HLH and carrying monoallelic mutations, hypomorphic variants, or defects in two different HLH-related genes (1–3). More recently, the term “sporadic HLH” has been applied to patients in whom, in spite of a high level of clinical suspicion, the currently available tests did not detect functional abnormalities (4). In most of these cases, the age of onset of disease is delayed, and the severity of the symptoms is reduced comparing with traditional FHL. Therefore, HLH is now portrayed as a “threshold” disease including a continuum of clinical presentations in which diverse etiologies and pathogenic mechanisms result in a similar end point of hemophagocytic syndrome.

## Case Description

Here, we describe a 9-month-old boy, born from nonconsanguineous parents, with past medical history of cutaneous Langerhans cell histiocytosis (LCH) diagnosed at 2 months of age. He was admitted to our institution with an exacerbation of cutaneous lesions, fever, vomit, hypoalbuminemia, diarrhea, and generalized edema; hence, an extended study of the disease is performed confirming the diagnosis of systemic LCH. Treatment was started with prednisone and vinblastine following the LCH-III international protocol, and after 1 month of disease progression, the patient started with Ara-C and cladribine (LCH-S 2005). After the third cycle of Ara-C and cladribine, the patient presented with a complete hematological remission of LCH. However, high ferritin levels (2,800 ng/mL) and triglycerides (280 mg/dl), bone marrow hemophagocytosis, low NK cytotoxicity, and elevated sCD25 levels were detected. As the patient fulfilled five of eight HLH diagnostic criteria (**Supplementary Table 1**), HLH treatment was included resulting in a control disease with a decrease in the CD25s levels and normalized ferritin levels, with recovery of NK cell cytotoxicity.

**Figure 1:**
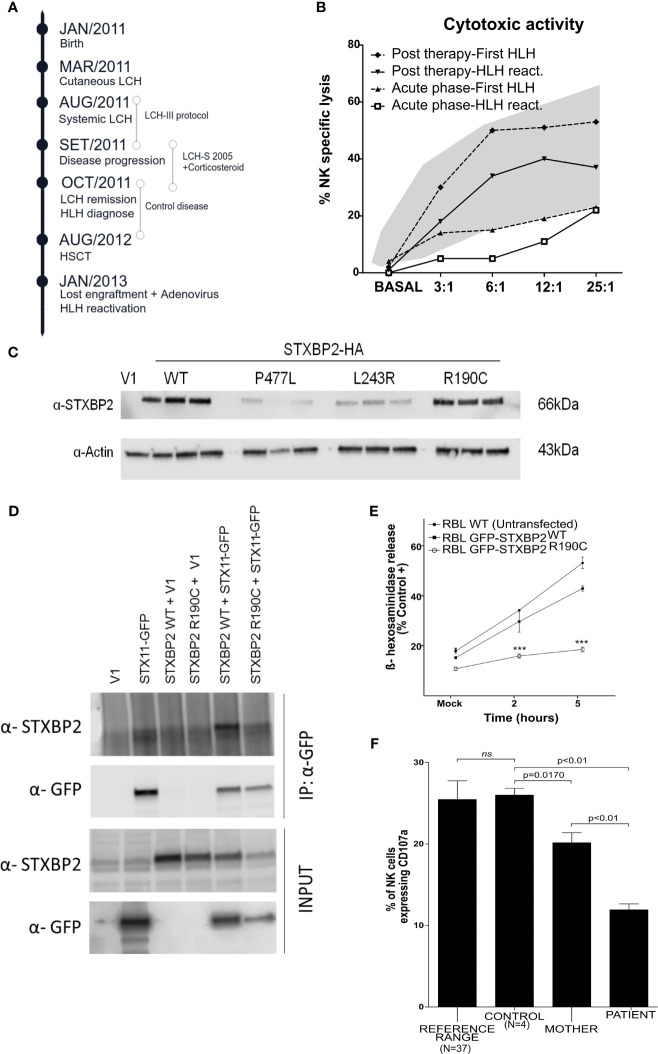
Analysis *in vitro* of the dominant negative mutation STXBP2-R190C. **(A)** Schematic timeline chronology of the patient illustrating the HLH onset, the HLH relapse, as well as the treatment used in each situation. **(B)** Cytotoxic activity during both HLH episodes (First HLH and HLH reactivation), during acute HLH phases (lower lines), and after HLH 2004 treatment (upper lines) in both episodes. The shaded area represents the mean values ±2 SD observed in healthy children (N=40). **(C)** COS-7 cells were transfected with an empty vector (V1) or with HA-tagged WT or mutant STXBP2 (L243R and P477L were used as negative controls). The expression levels were determined by western blot. **(D)** COS-7 cells were cotransfected with plasmids as indicated in the figure. After 24 h, cell lysates were immunoprecipitated with anti-GFP antibody and assessed for the presence of STXBP2 by western blot to proof the interaction with STX11-GFP. **(E)** RBL-2H3 cells were stably transfected with STXBP2-WT-EGFP or STXBP2-R190C-EGFP and were stimulated with PMA (5 ug/ml) and Ionomycin (1mM) for 2 and 5 h. The data shown are the mean ± SEM of two independent experiments each performed in triplicate (***p < 0.001). **(F)** Percentage of unmanipulated NK cells expressing CD107a after K562 stimulation from the patient, his mother, a healthy control, and 37 pediatric healthy donors used to show the reference range we use in our daily routine. The data shown are the mean ± SEM. (*ns; 0.3304)*.

Due to LCH progression, the patient (16 months of age) received allogeneic transplant (donor father), being referred again (5 months later) with loss of the chimera, Adenovirus infection, and hemophagocytic syndrome. Then, the patient required multiple treatments including cyclosporine, vp16, corticosteroids, gammaglobulins, ribavirin, cidofovir, and finally alemtuzumab, with control of viral reactivation and secondary hemophagocytic (March 2013 receives last dose of alemtuzumab), currently out of treatment, asymptomatic, without active disease (**Figure 1A**).

During the loss of chimera episode and HLH reactivation, impaired NK cytotoxicity was observed with a recovery after treatment (**Figure 1B**). The direct sequencing of the genes involved in primary HLH (*PRF1*, *UNC13D*, *STXBP2*, *STX11*, and *RAB27A*) only revealed one low-frequency (MAF=0.0005) maternally inherited heterozygous variant in the *STXBP2* gene (c.568C>T/p.R190C). The study included primers for sequencing flanking regions of up to 20 nucleotides, where intronic changes with functional consequences are usually located, excluding alterations such as mutations in intron 1 of the *UNC13D* gene. To rule out any other potential pathogenic defects associated to HLH, we performed an NGS targeted gene panel containing 427 PID-causing genes (see full gene list in **Supplementary Material**). Results confirmed the presence of the R190C heterozygous variant in the *STXBP2* gene. No additional pathogenic mutations in HLH-associated genes or in other genes related with the clinical phenotype of our patient were found. STXBP2-R190C has been described (3) and recently characterized (5) leading to a severe deleterious effect on STXBP2 function. We performed a careful clinical and molecular evaluation of the patient providing additional arguments for the STXBP2-R190C pathogenicity.

## Methods

Patient PBMCs were stimulated *ex vivo *with the K562 cell line to study NK cell cytotoxicity and degranulation. Moreover, we transfected COS-7 cells to analyze the STXBP2-R190C expression and to test the interaction with STX11. Finally, the RBL-2H3 cell line expressing STXBP2-WT-EGFP or R190C-EGFP was used for degranulation assays. Detailed information on the experiments and reagents are described in the **Supplementary Material**.

## Results and Discussion

The affected residue R190 is strongly conserved across evolution (**Supplementary Figure 1**), and computational analysis of the 3D structure revealed a rich interaction network important for stabilizing the STXBP2 protein domain 2 (**Supplementary Table 2**). To investigate the effect of the *STXBP2* monoallelic mutation R190C, we determined protein expression levels by western blot in transfected COS-7 cells (constructs STXBP2-WT and STXBP2-R190C), demonstrating that the mutation STXBP2-R190C does not affect protein expression. Two described mutations (L243R and P477L) with absent/low expression of STXPB2 have been included as pathological controls (**Figure 1C**) (6, 7).

To study the pathologic role of this mutation and specifically the ability to interact with STX11, the COS-7 cell line was transiently cotransfected with the HA-STXBP2-R190C and GFP-STX11-WT. Co-immunoprecipitation experiments of HA-STXBP2-WT with GFP-STX11-WT showed a band at 66 kDa when using the anti-STXBP2 antibody, indicating a normal interaction between proteins demonstrating that STXBP2-R190C maintains the ability to interact with STX11 (**Figure 1D**). Finally, to examine whether this mutation had a functional effect on the protein, the experimental conditions were adjusted to study the degranulation capacity in the RBL-H3 cell line transfected with EGFP-STXBP2-R190C or WT constructions (**Supplementary Material** and **Supplementary Figure 2**). RBL-2H3 cells stably expressing EGFP-STXBP2-R190C showed a significant reduced degranulation activity compared to EGFP-STXBP2-WT and nontransfected RBL-2H3 cells (**Figure 1E**). To explore the effect of the STXBP2-R190C mutation on the patient’s NK cells in a healthy state, PBMCs were isolated from the patient, his mother (heterozygous carrier), and a healthy donor. PBMCs were incubated 2 h with K562 cells at a ratio 1:1 to measure the upregulation of CD107a on NK cells. NK cells expressing CD107a (capable of degranulation) were significantly reduced in both the patient’s and patient’s mother’s NK cells compared to a healthy control group (n=4) (**Figure 1F**). Thus, degranulation of unmanipulated NK cells was reduced even without any inflammatory episodes and in a stable situation. It is possible that the reduced degranulation demonstrated in this patient allows adequate cytotoxic function under homeostatic conditions. However, under external inflammatory/infectious stimuli, it is plausible that, particularly in pediatric patients whose immune system is not fully competent, this function may not allow adequate control of an infectious or inflammatory response, resulting in HLH.

The evaluation of patients with these characteristics opens up the discussion of how to assess cases with monoallelic mutations in HLH-related genes and whether we should consider these patients as an HLH high-risk group. In addition, a functional characterization of monoallelic variants is essential not only to make the diagnosis more reliable but also to provide appropriate genetic counseling to families. Published series of patients with HLH with monoallelic mutations in HLH-related genes have revealed the difficulty of interpreting these genetic variants as well as their relevance in pathogenesis. Here, we have studied the role of the monoallelic STXBP2-R190C mutation found in a patient with two flares of HLH, one in the context of Langerhans cell histiocytosis disease and the other with Adenovirus infection. Using RBL-H3 as a cellular model, we have shown that the STXBP2-R190C mutation shows a dramatic decrease in degranulation. However, it is particularly striking to find a recovery of NK cytotoxic function after HLH therapy. HLH development has been described as a threshold disease depending on the trigger and residual cytotoxic capacity of NK (8). Thus, we speculate that the interaction of the STXBP2/STX11 complex (not affected by the STXBP2-R190C mutation) may not be adequate when a higher demand is required (e.g., viral infections, LCH). This hypothesis is in line with the descriptions of a possible novel binding mode of the domain 2 of the STXBP2 complex with other factors that could be truncated by the mutation (5).

The significant differences in degranulation between the mother and the patient and the fact that the mother has not experienced any HLH/MAS events during her lifetime led to the speculation that additional factors (e.g., age at episode onset, trigger, genetic background, and environment) are involved in the development of HLH. This is consistent with other publications (1, 5, 9) in which relatives of patient cases who were carriers of monoallelic variants had not yet manifested with HLH despite reduction in NK cell function.

We know our limitations, and we are conscious that the results provided by using RBL-2H3 (rat basophilic cell line) for the functional studies may be discussed. However, we chose this model for the following reasons: First, because in this cell line, STXBP2 has been shown to participate in the degranulation process through the IgE receptor (10). Second, this model has been used to study the essential regulatory role of UNC13D in secretory granule control (11). Last but not least, the STXBP2 protein has a high homology between humans and rats (≥94%). Following in this line, since the RBL-2H3 cell line is able to degranulate under untransfected conditions because it expresses WT protein, we assumed a heterozygosity scenario when transfected with the STXBP2-R190C mutation. Finally, we consider that the functional and molecular characterization of STXBP2-R190C monoallelic mutation would indicate the recommendation of regular medical follow-up, especially in pediatric carrier patients. It is therefore useful to achieve an extensive functional and genetic characterization of HLH patients with diverse feature to ascertain the underlying defects and consequently future therapeutic implications.

Therefore, it is useful to achieve a comprehensive functional and genetic characterization of HLH patients to determine the underlying defects and, consequently, to learn whether the defects involve a potential risk factor with implications for patient follow-up and treatment.

## Data Availability Statement

The raw data supporting the conclusions of this article will be made available by the authors, without undue reservation.

## Ethics Statement

Ethical review and approval was not required for the study on human participants in accordance with the local legislation and institutional requirements. Written informed consent to participate in this study was provided by the participants’ legal guardian/next of kin.

## Author Contributions

LV-G: Performed the research design, data analysis, clinical data collection, and writing the manuscript. RR: Participated in performing the research and data analysis. RC: Molecular study design and mutation analysis. XC: Contributed with the mutation analysis. VC, JD, and LA: Patient care, clinical data collection, and critically reviewed the manuscript. JS and MM-G: Design and conducting the study, analysis of the results, and writing and reviewing the manuscript. All authors contributed to the article and approved the submitted version.

## Funding

This work was funded by the Instituto de Salud Carlos III, grants PI17/00660 and PI18/00346, co-financed by the European Regional Development Fund (ERDF).

## Conflict of Interest

The authors declare that the research was conducted in the absence of any commercial or financial relationships that could be construed as a potential conflict of interest.

## Publisher’s Note

All claims expressed in this article are solely those of the authors and do not necessarily represent those of their affiliated organizations, or those of the publisher, the editors and the reviewers. Any product that may be evaluated in this article, or claim that may be made by its manufacturer, is not guaranteed or endorsed by the publisher.

## Supplementary Material

Supplementary Figure 1 Multiple sequence alignment (MSA) of various species for *STXBP2, STXBP1* and *STXBP3* ordered according to sequence identity (Seq. Ident.) with respect to *STXBP2* of *Homo sapiens*. Conserved residues at position 190 are indicated with an arrow.

Supplementary Figure 2 Flow cytometry histogram confirming consistent EGFP expression (blue lines) in RBL-2H3 cell line. Untransfected RBL-2H3 (grey) cells were used to set the gates.
