# Supplementary material for: Case Report: Characterizing the Role of the STXBP2-R190C Monoallelic Mutation Found in a Patient With Hemophagocytic Syndrome and Langerhans Cell Histiocytosis
Source: Front Immunol. 2021 Sep 23;12:723836. doi: 10.3389/fimmu.2021.723836 (PMC8496341; doi:10.3389/fimmu.2021.723836)
Supplement: Supplementary Figure 1 — Multiple sequence alignment (MSA) of various species for STXBP2, STXBP1 and STXBP3 ordered according to sequence identity (Seq. Ident.) with respect to STXBP2 of Homo sapiens. Conserved residues at position 190 are indicated with an arrow. [file DataSheet_1.docx]

**Supplementary Figures and Tables**

Supplementary Figure 1

Supplementary Figure 2


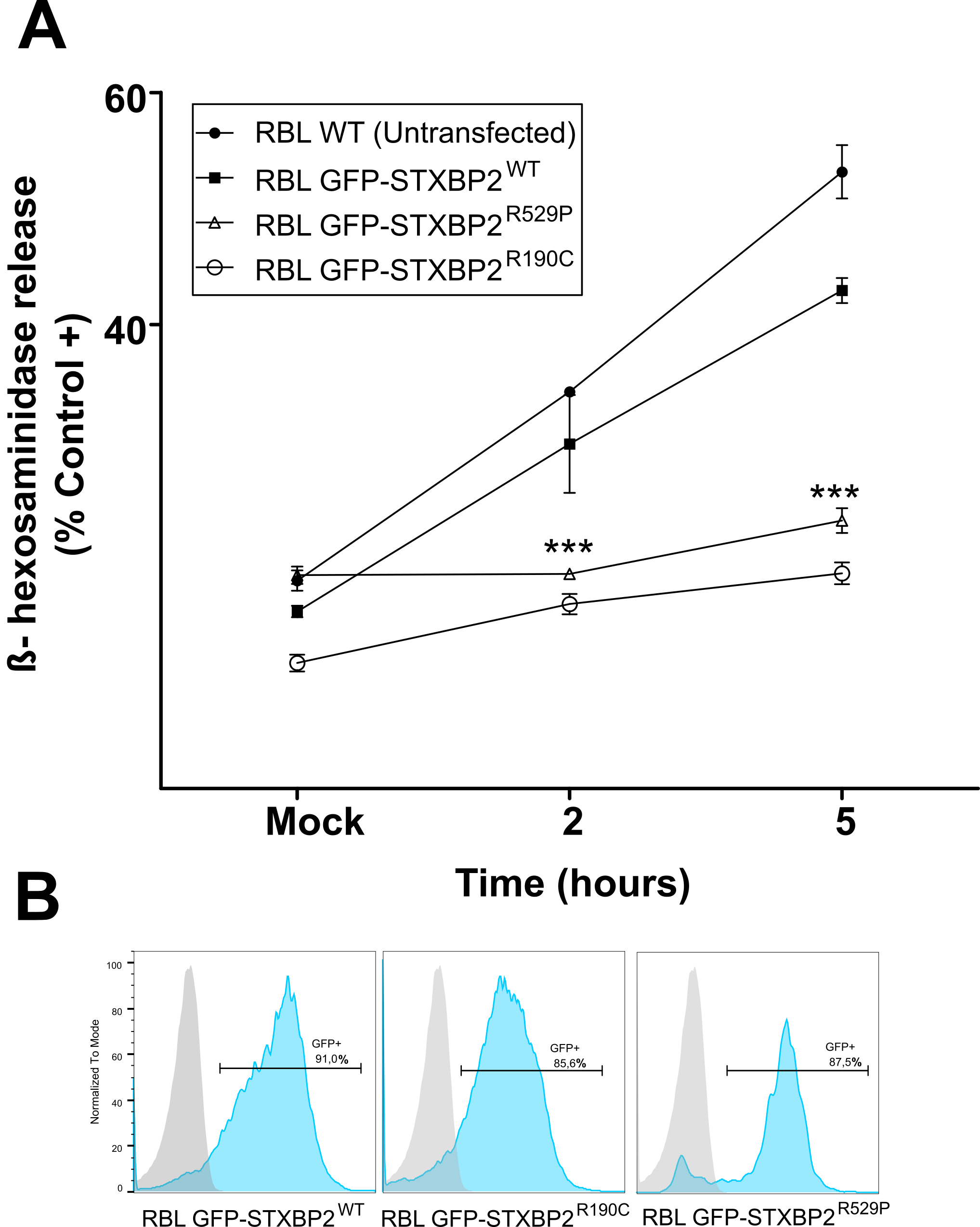


Supplementary Table 1. Clinical description and laboratory findings of the patient.

| **Clinical information** |  |
| --- | --- |
| Ethnic Origen | Caucasian |
| Consanguinity | No |
| Sex | M |
| Onset of disease | 9 months |
| Trigger | EBV |
| Fever | No |
| Splenomegaly | No |
| **Laboratory tests** |  |
| Haemoglibin (9-12,2g/dL) | Normal |
| Platelets (150-670x10^9/L) | Normal |
| Neutrophils (1,4-6,5x10^9/L) | Normal |
| Triglycerides (60-100mg/dL) | **280** |
| Fibrinogen (2,38-4.98g/dL) | **0.9** |
| Ferritin (25-400ng/mL) | **2800** |
| AST (5-40UI/L) | 19 |
| ALT (5-40UI/L) | 41 |
| Bone Marrow Haemophagocytosis | **Yes** |
| CD25s ( <2400UI/mL ) | **6224** |
| Perforin expression | Normal |
| NK degranulation | Reduced |
| Cytotoxicity | **Absent** |
| Central Nervous System affectation | No |
| HLH flares/ time after the first episode | Yes/ 2 years |
| Fulfilment of HLH diagnostic criteria | Yes (5/8) |
| Disease recurrence | Yes |
| ALT, Alanine transaminase; AST, Aspartate transaminase; CD25s, soluble form of alpha subunit of interleukin-2 receptor; EBV, Ebstein-Barr Virus. | |

Supplementary Table 2. List of amino acids predicted to interact with R190 at the STXBP2 protein.

| Residue | Minimum Distance (Å) | Nº of Contacts | Domain |
| --- | --- | --- | --- |
| Phe498 | 3.235 | 32 | 2 |
| Ile189 | 1.329 | 30 | 2 |
| Leu231 | 3.603 | 16 | 2 |
| Trp496 | 2.913 | 15 | 2 |
| Leu230 | 2.827 | 13 | 2 |
| Ile232 | 2.911 | 12 | 2 |
| Pro497 | 3.283 | 11 | 2 |
| Gln229 | 3.287 | 8 | 2 |
| Leu491 | 3.835 | 7 | 2 |
| Ala188 | 3.868 | 5 | 2 |
| Val499 | 4.880 | 3 | 2 |
| Ala202 | 4.636 | 2 | 2 |
| Arg493 | 4.841 | 1 | 2 |

Supplementary List:

List of 427 PID-associated gens included in our NGS targeted gene panel.

ACP5, ACTB, ACD, ADA, ADA2, ADAM17, ADAMTS13, ADAR, AICDA, AIRE, AK2, ALPI, ANGPT1, AP1S3, AP1S3, AP3B1, AP3D1, APOL1, ARHGEF1, ARPC1B, ATM, ATP6AP1, B2M, BACH2, BCL10, BCL11B, BLM, BLNK, BLOC1S6, BTK, C1QA, C1QB, C1QC, C1R, C1S, C2, C3, C4A, C4B, C5, C6, C7, C8A, C8B, C8G, C9, CARD11, CARD14, CARD9, CARMIL2, CASP10, CASP8, CCBE1, CD19, CD247, CD27, CD3D, CD3E, CD3G, CD40, CD40LG, CD46, CD48, CD55, CD59, CD70, CD79A, CD79B, CD81, CD8A, CDC42, CDCA7, CEBPE, CFB, CFD, CFH, CFHR1, CFHR3, CFHR4, CFHR5, CFI, CFP, CHD7, CIB1, CIITA, CLCN7, CLEC7A, CLPB, COPA, CORO1A, CR2, CSF2RA, CSF2RB, CSF3R, CTLA4, CTPS1, CTSC, CXCR4, CYBA, CYBB, CYBC1, DBF4, DBR1, DCLRE1B , DCLRE1C, DEF6, DGAT1, DGKE, DKC1, DNAJC21, DNASE1L3, DNASE2, DNMT3B, DOCK2, DOCK8, EFL1, ELANE, EPG5, ERBIN, ERCC6L2, EXTL3, F12, FAAP24, FADD, FAS, FASLG, FAT4, FCGR1A, FCGR2A, FCGR2B, FCGR3A, FCGR3B, FCGRT, FCHO1, FCN3, FERMT1, FERMT3, FOXN1, FOXP3, FPR1, G6PC3, G6PD, GATA2, GFI1, GINS1, HAVCR2, HAX1, HELLS, HMOX1, HTR1A, HYOU1, ICOS, ICOSLG, IFIH1, IFNAR1, IFNAR2, IFNG, IFNGR1, IFNGR2, IGHM, IGLL1, IKBKB, IKBKG, IKZF1, IL10, IL10RA, IL10RB, IL12B, IL12RB1, IL12RB2, IL17F, IL17RA, IL17RC, IL18BP, IL1RN, IL21, IL21R, IL23R, IL2RA, IL2RB, IL2RG, IL36RN, IL6R, IL6ST, IL7R, INO80, IRAK1, IRAK4, IRF2BP2, IRF3, IRF4, IRF7, IRF8, IRF9, ISG15, ISG15, ITCH, ITGB2, ITK, ITPKB, ITPR3, JAGN1, JAK1, JAK2, JAK3, KDM6A, KMT2A, KMT2D, KRAS, LACC1 , LAMTOR2, LAT, LCK, LIG1, LIG4, LPIN2, LRBA, LRRC8A, LYST, MAGT1, MALT1, MAP3K14, MASP2, MBL2, MCM4, MEFV, MOGS, MPO, MRTFA, MS4A1, MSH6, MSN, MTHFD1, MVK, MYD88, MYOF, MYSM1, NBAS, NBN, NCF1, NCF2, NCF4, NCKAP1L, NCSTN, NEIL3, NFAT5, NFE2L2, NFKB1, NFKB2, NFKBIA, NFKBID, NHEJ1, NHP2, NLRC4, NLRP1, NLRP12, NLRP3, NOD2, NOP10, NOS2, NRAS, NSMCE3, OAS1, ORAI1, OSTM1, OTULIN, PARN, PAX5, PEPD, PGM3, PIK3CD, PIK3R1, PLCG2, PLEKHM1, PLG, PMS2, PNP, POLA1, POLD1, POLD2, POLE, POLE2, POLR3A, POLR3C, POLR3F, POMP , POU2AF1, PRF1, PRKCD, PRKDC, PSEN1, PSENEN, PSMA3 , PSMB10 , PSMB4 , PSMB8, PSMB9 , PSMG2, PSTPIP1, PTEN, PTPRC, RAB27A, RAC2, RAG1, RAG2, RANBP2, RASGRP1, RBCK1, RC3H1, RECQL4, REL, RELA, RELB, RFX5, RFXANK, RFXAP, RHOG, RHOH, RIPK1, RMRP, RNASEH2A, RNASEH2B, RNASEH2C, RNF168, RNF31, RNU4ATAC, RORC, RPSA, RTEL1, SAMD9, SAMD9L, SAMHD1, SASH3, SBDS, SEC61A1, SEMA3E, SERPING1, SH2D1A, SH3BP2, SH3KBP1, SKIV2L, SLC29A3, SLC35C1, SLC37A4, SLC39A7, SLC46A1, SLC7A7, SMARCAL1, SMARCD2, SNX10, SOCS1, SP110, SPINK5, SPPL2A, SRP54, STAT1, STAT2, STAT3, STAT5B, STIM1, STK4, STN1, STX11, STXBP2, TAP1, TAP2, TAPBP, TAZ, TBK1, TBX1, TBX21, TCF3, TCIRG1, TCN2, TERC, TERT, TFRC, TGFB1, TGFBR1, TGFBR2, THBD, TICAM1, TINF2, TIRAP, TLR3, TLR8, TMC6, TMC8, TMEM173, TNFAIP3, TNFRSF11A, TNFRSF13B, TNFRSF13C, TNFRSF1A, TNFRSF4, TNFRSF9, TNFSF11, TNFSF12, TOM1, TOP2B, TPP2, TRAC, TRAF3, TRAF3IP2, TREX1, TRIM22, TRNT1, TTC37, TTC7A, TYK2, UBA1, UNC13D, UNC93B1, UNG, USB1, USP18, VPS13B, VPS45, WAS, WASF2, WDR1, WIPF1, WRAP53, XIAP, ZAP70, ZBTB24, ZNF341.
